# The Dynamic Change of Immune Responses Between Acute and Recurrence Stages of Rodent Malaria Infection

**DOI:** 10.3389/fmicb.2022.844975

**Published:** 2022-02-17

**Authors:** Suilin Chen, Yuanli Gao, Yongling Fan, Shuai Guo, Jian Zhou, Taiping Liu, Wenyue Xu

**Affiliations:** ^1^Department of Pathogenic Biology, Army Medical University (Third Military Medical University), Chongqing, China; ^2^Key Laboratory of Extreme Environmental Medicine, Ministry of Education of China, Chongqing, China; ^3^Institute of Immunology, Army Medical University (Third Military Medical University), Chongqing, China

**Keywords:** malaria parasite, scRNA-seq, recurrence, adaptive immune responses, innate immune responses

## Abstract

Malaria infections are persistent as frequent recrudescence of the disease may occur following the acute infection stage, but the different immune responses that control the acute and recrudescence stages are still largely unknown. Using single-cell RNA sequencing (scRNA-seq), we showed that the number of Th1 and plasma cells in the spleen was significantly reduced during the recurrence stage compared to the acute stage of *Plasmodium chabaudi chabaudi AS* (*P. chabaudi*) infection. Additionally, the ability of both CD4^+^ T cell responses and B cells to control *P. chabaudi* recurrence was significantly reduced compared to their roles in the control of acute infection. In contrast, the number of innate immune cells, including red pulp macrophages (RPMs), gamma delta (γδ) T cells, and Dendritic cells (DCs) were significantly increased during the recurrence stage and showed to be critical for *P. chabaudi* infection recurrence control. Thus, our data strongly suggest the complementary role of innate immune responses in controlling malaria recrudescence when adaptive immune responses are suppressed. These findings shed new light on the development of immune interventions against malaria.

## Introduction

Malaria, one of the most devastating diseases worldwide, is caused by *Plasmodium* infection. Acute blood-stage malaria is controlled by parasite-specific CD4^+^ T cell responses (Pombo et al., 2002), antibodies (Cohen et al., 1961), and CD8^+^ T cell responses (Junqueira et al., 2018). However, some parasite clones are able to evade the host immune attack by switching their variant antigens, such as *var*, repetitive interspersed family (*rif*), and subtelomeric variant open reading frame (*stevor*), resulting in frequent recrudescence (Scherf et al., 2008). Thus, after the acute infection stage, malaria infection persists at low levels for several months or years, and this has been regarded as a reservoir for malaria transmission (Ouedraogo et al., 2009; Okell et al., 2012; Churcher et al., 2015; Barry et al., 2021). Understanding the underlying mechanisms involved in the host control of malaria recrudescence will provide us with novel clues for designing immune intervention measures against malaria parasites and blocking malaria transmission.

*Plasmodium chabaudi* (*P. chabaudi*, a rodent malaria parasite) infection is characterized by an acute infection stage (before day 10) that can subsequently persist for 2 to 3 months with frequent recurrences of infection (Stephens et al., 2012). The frequent recurrence of *P. chabaudi* infection, which is similar to that of recrudescence of human malaria, is thus responsible for persistent infection after the acute infection stage. Therefore, *P. chabaudi* infection is a well-established model for studying the mechanisms underlying the immune control of malaria recrudescence (Mamedov et al., 2018). The recurrence of *P. chabaudi* infection has been attributed to differential expression of variant-like genes during the acute and recurrence stages (Brugat et al., 2017), but the predominant immune effectors that control the recurrence are largely unknown.

It is well known that both parasite-specific CD4^+^ T cell responses and antibodies are critical for the control *P. chabaudi* infection during the acute phase (Meding and Langhorne, 1991). In contrast, during the chronic infection stage, both CD4^+^ T and CD8^+^ T cells tend to be exhausted with upregulated expression of programmed cell death-1 (PD-1) and lymphocyte-activation gene 3 (LAG3) (Butler et al., 2012; Illingworth et al., 2012; Horne-Debets et al., 2013), implying that the immune system is impaired and cannot control the chronic infection. Furthermore, a recent study demonstrated that γδT cells, which are significantly increased in both human malaria and *P. chabaudi* chronic infection, are involved in the control of *P. chabaudi* recurrence through the secretion of macrophage colony-stimulating factor (M-CSF) (Mamedov et al., 2018). Moreover, the loss or dysfunction of γδT cells has been closely associated with clinical immunity in *P. falciparum* infected children (Jagannathan et al., 2014), indicating the importance of γδT cells in the control of chronic malaria infection. These findings strongly suggest that the immune effectors involved in the control of *P. chabaudi* recurrence might be different from those involved in the control of acute infection.

In the current study, we revealed the dynamic change in host immune responses against *P. chabaudi* infection in the acute phase and parasitemic recurrence stage using single-cell RNA sequencing (scRNA-seq). In contrast to their important role in the control of acute phase *P. chabaudi* infection, CD4^+^ T cells and antibodies are less critical than the innate immune responses in controlling *P. chabaudi* recurrence. Strikingly, innate immune responses were found to be essential for the control of the recurrence of *P. chabaudi.*

## Materials and Methods

### Mice and Experimental Malaria Model

C57BL/6J mice (6–8 wk of age) were purchased from the Animal Institute of the Academy of Medical Science (Beijing, China). B cell-deficient mice (μMT, C57BL/6 background) were originally obtained from the Jackson Laboratory (Bar Harbor, ME, United States). All mice were bred and maintained in our pathogen-free animal facility. Only female mice were used in the current study. All mouse studies were reviewed and approved by the Animal Ethics Committee of the Army Medical University (Third Military Medical University) Institute of Medical Research, under the approval number AMUWEC2020135. *P. chabaudi* was maintained in our laboratory. For infection studies, mice were inoculated intraperitoneally with 1 × 10^6^ parasitized RBCs (pRBCs). Parasitemia was determined by examination of Giemsa-stained thin blood smears every other day after infection.

### Single-Cell RNA Sequencing, Library Preparation, and Alignment

For splenocytes isolation, mouse spleens were mechanically disrupted with the back of a 10 ml syringe, filtered through a 70 μm strainer, and subjected to red blood cell lysis with 1 × red blood cell lysis solution (Miltenyi Biotec, Bergisch Gladbach, Germany). Cells were washed twice with cold Roswell Park Memorial Institute (RPMI) 1,640 medium supplemented with 2 μM glutamine, 100 U ml^–1^ penicillin/streptomycin and 5–10% fetal bovine serum (FBS). Trypan blue staining showed a >90% viability of splenocytes. For scRNA-seq analysis, splenocytes isolated from *P. chabaudi*-infected mice (*n* = 5) at day 8 and day 16 post-infection, were loaded on a Chromium Controller (10 × Genomics, Pleasanton) at a concentration of 10^3^ cells/μl per sample, to generate Gel Bead-In EMulsions (GEMs). Barcoded sequencing libraries were constructed using the Chromium Single Cell 3′ Reagent Kits v3 (10 × Genomics) according to the manufacturer’s instructions. Following library preparation, sequencing was performed with paired-end sequencing of 150 nt at each end using one lane of the NovaSeq 6000 per sample. Raw sequencing data were processed using Cell Ranger (10 × Genomics; v6.0.1). The CellRanger function “count” was used to align raw reads with the STAR aligner against the mm10 reference genome. After counting non-redundant unique molecular identifiers (UMIs), single-cell digital-expression matrices were obtained. Library preparation and sequencing were performed at Jiayin Biotechnology Ltd. (Shanghai, China).

### Single-Cell Clustering Using Seurat

The output “cellranger” matrices were loaded into the Seurat package (v4.0.3) in R (v4.0.3). To remove low-quality cells, cells with unique gene counts (less than 200) and genes expressed in fewer than three cells were filtered out. Standard data pre-processing was performed to remove mitochondrial genes, residual red blood cells, cell doublets, and multiplets. The UMI count matrix for each cell was normalized using a global scaling normalization method “LogNormalize.” The “ScaleData” function implemented in the Seurat package was used to remove the unwanted sources of variation through the “vars.to.regress” argument. The 3,000 most variable genes were selected for further analysis. Before dimensionality reduction, the data were scaled such that the average expression was 0, and the variance was equal to 1. The first 10 dimensions of the principal component analysis (PCA) were used for cluster identification, and the clusters were identified using the FindClusters function with a resolution of 0.5. Datasets of multiple samples were combined into a single dataset using canonical correlation analysis with the “IntegrateData” function. Uniform manifold approximation and projection (UMAP) analysis was used to visualize the selected datasets. General cell types were annotated based on the cell type-specific marker gene expression and the genes driving heterogeneity of clusters (*Cd79a*, *Cd79b*, B cells; *Cd3e*, T cells; *Flt3*, DCs; *Itgam*, *Adgre1*, monocytes/macrophages; *Klra8*, NK cells; *Ly6g*, neutrophils; *Pf4*, platelets). Immune cell subpopulations were annotated using previously reported genes to identify the following populations: CD4^+^ T cells (*Cd3e*, *Cd4*), CD8^+^ T cells (*Cd3e*, *Cd8a*, *Cd8b1*), γδ T cells (*Cd3e*, *Tcrg-C1*, *Tcrg-C2*), NKT cells (*Cd3e*, *Klrb1c*, *Klrc1*), T helper type 1 (Th1) cells (*Cd3e*, *Tbx21*, *Ifng*), T follicular-helper (Tfh) cells (*Cd3e*, *Bcl6*, *Cxcr5*), T-regulatory (Treg) cells (*Cd3e*, *Foxp3*, *Il2ra*), naive CD8^+^ T cells (*Cd3e*, *Cd8a*, *Cd8b1*, *Ccr7*, *Il7r*), effector CD8^+^ T cells (*Cd3e*, *Cd8a*, *Cd8b1*, *Gzma*, *Prf1*), plasma cells (*Cd79a*, *Cd79b*, *Jchain*, *Sdc1*), GC B cells (*Cd79a*, *Cd79b*, *Fas*, *Ighd*).

### Differential Gene Expression Analysis

The Seurat package in R (v4.0.3) was used to perform differential gene expression analysis and compare the expression profiles between the two groups. Differential expression analysis was conducted using the default two-sided non-parametric Wilcoxon rank-sum test with Bonferroni correction, using all genes in the dataset. *P*-values were adjusted for multiple testing using the false discovery rate (FDR). Genes with a *P*-value of < 0.05 was considered differentially expressed genes. The enhanced volcano plots were used to display the differentially expressed genes between the two cell clusters. Genes of interest were labeled.

### Flow Cytometry Analysis

The following antibodies were used for flow cytometry analysis: anti-mouse CD45R/B220 (APC, clone RA3-6B2, BioLegend), anti-mouse CD3 (FITC, clone 17A2, BioLegend), anti-mouse NK1.1 (PE, clone PK136, BioLegend), anti-mouse CD11c (APC, clone N418, BioLegend), anti-mouse CD11b (Percp/Cy5.5, clone M1/70, BioLegend), anti-mouse Ly6G (APC, clone 1A8, BioLegend), anti-mouse F4/80 (FITC, clone BM8, BioLegend), anti-mouse Ly6C (PE, clone HK1.4, BioLegend), anti-mouse CD11c (APC/Cyanine7, clone N418, BioLegend), anti-mouse TCRγ/δ (APC, clone GL3, BioLegend), anti-mouse CD3 (PE/Cyanine7, clone 17A2, BioLegend), anti-mouse CD4 (FITC, clone GK1.5, BioLegend), anti-mouse CD8α (APC, clone 53–6.7, BioLegend), anti-mouse CD45R/B220 (Pacific Blue, clone RA3-6B2, BioLegend), anti-mouse CD19 (PerCP/Cyanine5.5, clone 6D5, BioLegend). The LIVE/DEAD™ Fixable Violet Stain kit (Invitrogen, Grand Island, NY, United States) was used to determine the viability of cells. For flow cytometry staining, spleens were collected on the appropriate days, and single-cell suspensions of splenocytes were prepared as described above. A 1 × 10^6^ splenocytes were first incubated with anti-CD16/CD32 antibodies (clone 93, BioLegend) to block Fc receptors and then incubated with the antibodies of interest for 45 min. The cells were analyzed on a FACSCanto II instrument (BD Biosciences, San Jose, CA, United States), and the data were analyzed with FlowJo software.

### *In vivo* Depletion Assays

For CD4 and CD8^+^ T cell depletion assay, 200 μg anti-CD4 mAb (clone GK1.5, BioXcell), anti-mouse CD8β (clone 53–5.8, BioXcell) or the corresponding isotype control IgG was injected intraperitoneally into *P. chabaudi*-infected mice at days -1, 1, 3, 5, 7 or days 14, 16, 18, 20, 22 after the challenge. Depletion of B cells was accomplished by intraperitoneal injection of 500 μg of anti-CD19 mAb (clone 1D3, BioXCell) and 500 μg of anti-B220 mAb (clone RA3.3A1/6.1, BioXCell) or the corresponding isotype control IgG at 12, 14, 16, 18, and 20 days post-infection. CD4^+^ T cells, CD8^+^ T cells and B cells depletions were verified by staining blood samples with anti-mouse CD4 (APC, clone GK1.5, BioLegend), anti-mouse CD8α (FITC, clone 53–6.7, BioLegend), anti-mouse CD19 (PerCP/Cyanine5.5, clone 6D5, BioLegend), and anti-mouse B220 (Pacific Blue™, clone RA3-6B2, BioLegend). For phagocytes depletion assay, 200 μl of clodronate liposomes (Liposoma BV) or control liposomes were injected intravenously into *P. chabaudi*-infected mice at days 14, 17, 20 after infections.

### Statistical Analysis

The data were analyzed using GraphPad Prism version 8.0 (GraphPad Software, La Jolla, CA, United States). Data are shown as the means ± SD unless otherwise stated. Non-parametric tests (Mann-Whitney test) and two-way ANOVA were used to determine the statistical significance between groups. *P*-values < 0.05 were considered statistically significant.

## Results

### Single Cell Analysis Reveals the Dynamic Change of Immune Cells Between Acute and Recurrence Stages

**FIGURE 1:**
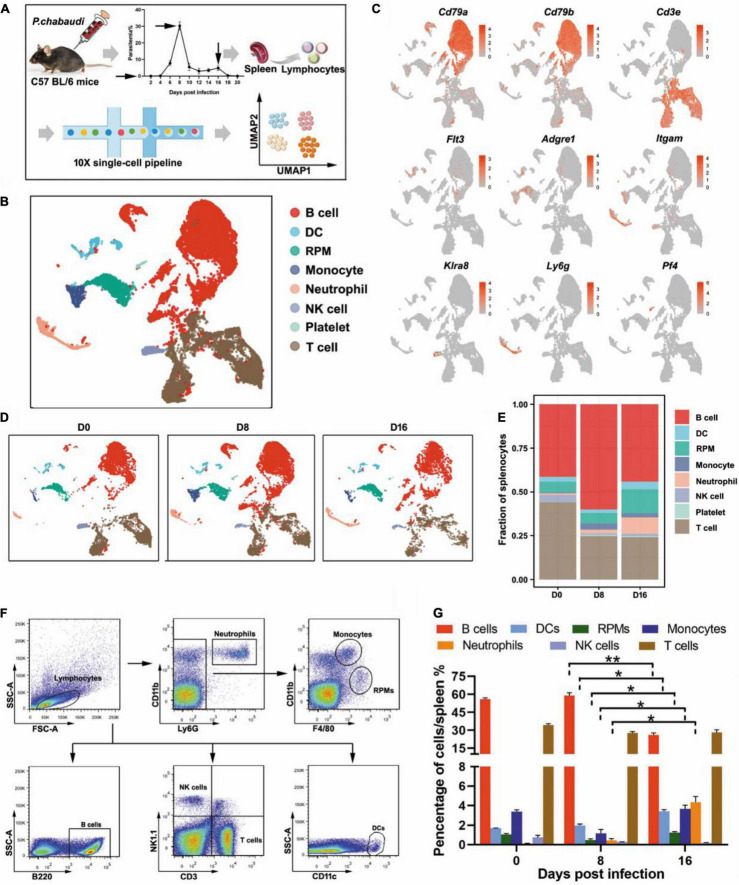
Single-cell analysis revealed the dynamic changes of immune cells between the acute and recurrence stages of *P. chabaudi* infection. **(A)** Schematics of the experimental pipeline. Splenocytes from five mice were pooled together at days 0, 8, and 16 post-infection for 10 × Genomics scRNA-seq. The arrow indicates the day when mouse spleens were harvested. **(B)** Unsupervised clustering was performed on the UMAP to identify groups of cells with similar gene expression. **(C)** UMAP visualization showing the expression of canonical immune cell markers in each cluster of cells. **(D)** UMAP plot from **(B)** split by the three time points post-infection. **(E)** Fractions of immune cell populations relative to all splenocytes as the immune cell numbers change during the *P. chabaudi* infection. **(F)** Representative flow cytometry analysis of major immune cell populations at days 0, 8, and 16 post-infection. **(G)** Cellular composition of immune cells evaluated by flow cytometry as in **(F)**. The data are presented as mean ± SD based on three independent biological replicates. Plasma cells were included in the population of B cells, *P < 0.05; **P < 0.01.

To investigate the predominant immune effectors that control the recurrence of rodent malaria, the splenic lymphocyte populations at the peak of the recurrent stage (day 16 post-infection) were compared to those at the peak of the acute infection stage (day 8 post-infection) using scRNA-seq (Figure 1A). UMAP analysis classified the splenic lymphocytes into eight clusters based on the cell-specific molecular markers, including T cells (*Cd3e*), B cells (*Cd79a*, *Cd79b*), NK cells (*Klra8*), DCs (*Flt3*), RPMs (*Adgre1*), monocytes (*Itgam*), neutrophils (*Ly6g*), and platelets (*Pf4*) (Figures 1B,C). The main lymphocytes were the B and T cells. However, a cluster of red blood cells was found in the initial UMAP analysis (data not shown), which was due to insufficient lysis of red blood cells during splenocyte preparation. The clusters of red blood cells were removed in the subsequent analysis.

All eight clusters were found in splenocytes collected from either uninfected mice or *P. chabaudi* infected mice at day 8 or day 16 post-infection, but the frequency of several clusters fluctuated significantly. Compared to baseline (day 0), the number of T cells decreased at day 8 post-infection but returned to the baseline level at day 16 post-infection. However, the number of B cells was significantly increased in the acute phase, but then significantly declined in the recurrence phase. In contrast, innate immune cells, including RPMs, DCs, and neutrophils, significantly increased during the recurrence stage compared to the acute stage (Figures 1D,E). The change trends of the infiltrated lymphocytes were well confirmed by flow cytometry analysis of splenocytes from *P. chabaudi*-infected mice at day 8 and day 16 post-infection (Figures 1F,G). Therefore, our single-cell analysis revealed significant changes in both adaptive and innate immune cells between the acute phase and the recurrence phase of *P. chabaudi* infection.

### CD4^+^ T Cell Responses Are More Efficient to Control Infection During the Acute Stage Than During the Recurrence Stage

It is well known that parasite specific CD4^+^ T cells are responsible for controlling blood-stage malaria infection (Meding and Langhorne, 1991). The acute infection of *P. chabaudi* drove the differentiation of Th0 into CD4^+^ Th1 cells and CD4^+^ Tfh cells (Lonnberg et al., 2017). Interferon-gamma (IFN-γ) secreted by Th1 cells then promoted macrophages to degrade phagocytosed parasites (Stephens et al., 2005), while CD4^+^ Tfh cells helped the B cells to generate antibodies (Perez-Mazliah et al., 2017). Additionally, acute malaria infection increased Treg cells, which reduce the CD4^+^ T cell response, to maintain immune hemostasis (Hisaeda et al., 2004; Nie et al., 2007; Haque et al., 2010). However, the dynamic change of CD4^+^ T cell responses in the whole process of *P. chabaudi* infection is still largely undefined.

**FIGURE 2:**
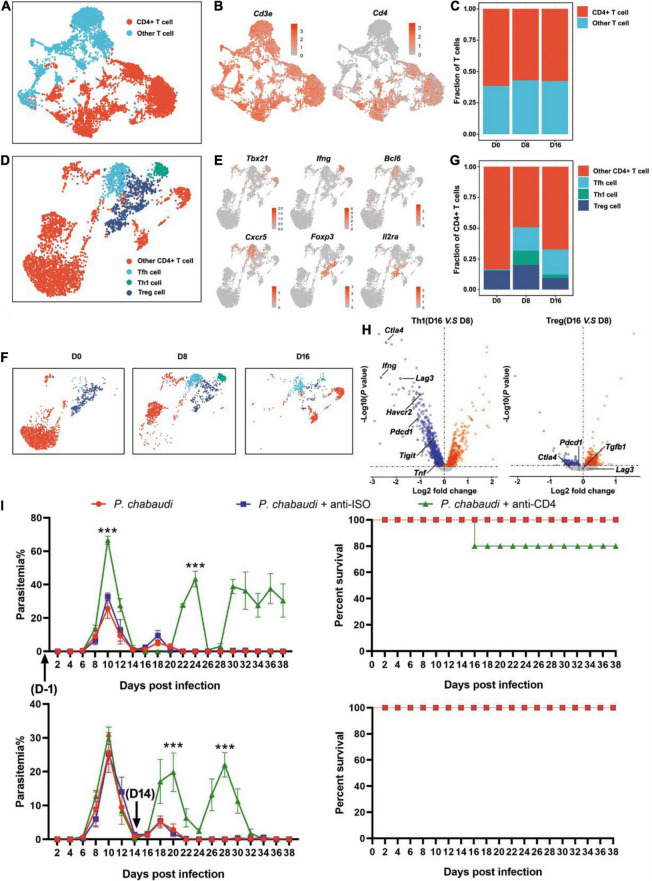
The functions of CD4^+^ T cells during the acute and recurrence stages of *P. chabaudi* infection. **(A)** UMAP projection plot of T cell populations merged from the three time points. **(B)** Feature plots depicting single-cell expression of canonical immune cell markers of CD4^+^ T cells. **(C)** Cell abundance frequencies relative to the total number of T cells at each time point. **(D)** UMAP plot of CD4^+^ T cells pooled together from the three time course samples. **(E)** Marker gene expression in CD4^+^ T cell clusters. **(F)** UMAP plot of CD4^+^ T cells (as in **D**) split into the three stages of infection. **(G)** Fraction of cells in each population relative to the total number of CD4^+^ T cells at each time point. **(H)** Volcano plots showing differential gene expression profiles between clusters of Th1 and Tfh cells at different time points, with a *P*-value of < 0.05. Blue represents downregulated genes and red represents upregulated genes. **(I)** Wild type (WT) mice (*n* = 5) were injected with anti-CD4 or control IgG on days –1, 1, 3, 5, 7 or days 14, 16, 18, 20, 22 after the challenge. All the mice were challenged with *P. chabaudi* on day 0, and the parasitemia and survival rate were monitored. All experiments were performed in triplicates and the data are presented as means ± SD, ***P < 0.001.

T cell population was firstly classified into clusters of CD4^+^ T cells (*Cd3e* and *Cd4*) and other T cells, according to the specific markers (Figures 2A,B). The frequencies of CD4^+^ T cells were comparable at the three time points (Figure 2C). Then, the CD4^+^ T cells were further divided into Th1 (*Cd3e*, *Tbx21*, and *Ifng*), Tfh (*Cd3e*, *Bcl6*, and *Cxcr5*), Treg (*Cd3e*, *Foxp3*, and *Il2ra*), and other CD4^+^ T cells, based on their specific markers (Figures 2D,E). Th1 cells were greatly increased during the acute stage as compared to the control, but then significantly decreased in the recurrent phase. An increased number of Tfh cells were also observed during the acute *P. chabaudi* infection stage, but no significant reduction in Tfh cells was observed during the recurrence stage. As expected, the number of Treg cells was also significantly increased during the acute stage and declined during the recurrence stage (to a level much lower than the baseline) (Figures 2F,G). Differential gene expression analysis showed that the signals of T cell exhausting markers, such as CTLA-4 (*Ctla4*), LAG3 (*Lag3*), Tim3 (*Harvcr2*), and PD-1 (*Pdcd1*) were significantly downregulated, but the signal of the main effector of Th1 cells, IFN-γ (*Ifng*), was also significantly reduced in Th1 cells in the recurrence phase, as compared to the gene expression in the acute phase (Figure 2H). No significant change of T cell exhausting markers in Treg cells was observed between acute stage and recurrence phase of *P. chabaudi* infection (Figure 2H). To compare the different roles of CD4^+^ T cell responses in the control of acute infection and recurrence of *P. chabaudi*, CD4^+^ T cells were depleted during the acute stage and before the onset of recurrence, respectively. After CD4^+^ T cells were depleted during the acute phase (Supplementary Figure 1), the parasitemia of *P. chabaudi* was greatly increased during this phase, and then fluctuated at high levels. The mice could not clear the parasite, and succumbed to the infection. In contrast, mice could clear the parasites after CD4^+^ T cells were depleted during the recurrence stage, although the depletion of CD4^+^ T cells also significantly elevated the *P. chabaudi* parasitemia (Figure 2I). Our data thus illustrate the more important role of CD4^+^ T cell response in the control of acute infection than in the control of recurrent infection of *P. chabaudi*, which was closely associated with the frequency change of CD4^+^ T cell subsets.

### Divergent Role of B Cells in the Control of Acute and Recurrent *Plasmodium chabaudi* Infection

With the help of Tfh cells, germinal center (GC) B cells were activated and then differentiated into memory B cells and long-lived plasma cells (LLPC) (Deenick and Ma, 2011). High-affinity antibodies were produced by the LLPC after the Ig type switch and destroyed malaria parasites through neutralization, activation of the complement system, mediation of antibody-dependent cellular cytotoxicity (ADCC) or opsonic phagocytosis mediated by complement fragments and antibodies (Chua et al., 2013; Julien and Wardemann, 2019). Using B cell-deficient mice (μMT) (Supplementary Figure 2), B cells were found to be necessary for the final elimination of the parasites but were not required for the control of early blood-stage malaria infection (von der Weid et al., 1994; Brugat et al., 2017). Using μMT mice in these studies, B cells were absent at the beginning of infection and thus their definitive role in the control of *P. chabaudi* recurrence was unknown.

**FIGURE 3:**
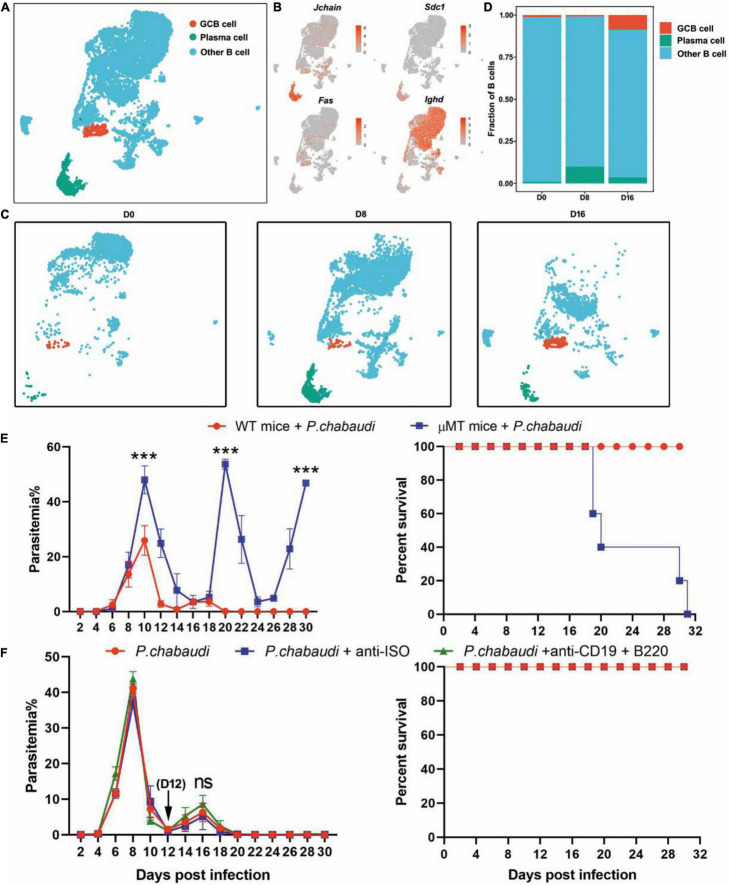
Divergent role of B cells in the control of acute and recurrent *P. chabaudi* infection. **(A)** UMAP projection and graph-based clustering of B cells pooled from all samples. **(B)** Expression of markers driving heterogeneity between B cell subtypes, including the GC B cells and plasma cells. **(C)** Visualization of UMAP plot from **(A)** split by samples during the three stages of infection. **(D)** Proportion of B cell populations relative to total number of B cells at each time point. **(E)** B cell-deficient mice and littermates (μMT, *n* = 5) were challenged intraperitoneal (i.p.) with 1 × 10^6^ pRBCs and the parasitemia and survival rate were recorded. **(F)** WT mice (*n* = 5) were injected with anti-B220 and anti-CD19 or control IgG on days 12, 14, 16, 18, 20 after the challenge. All experiments were performed in triplicates and the data are presented as means ± SD. ns, not significant; ***P < 0.001.

Therefore, we further classified splenic B cells into GC B (IgD^–^ FAS^+^) and plasma cells according to their specific markers (*Cd79a, Cd79b, Jchain, Sdc1*, plasma cells; *Cd79a, Cd79b, Fas, Ighd*, GC B cells) (Figures 3A,B). GC B cells were significantly increased during the recurrence stage, but not during the acute stage. In contrast, the number of plasma cells greatly increased in the acute phase, but sharply declined to a level slightly higher than that of the baseline, in the recurrence phase (Figures 3C,D). Consistently, the expression levels of either *Ighg2b* (IgG2b), *Ighg2c* (IgG2c) or *Ighg3* (IgG3) were significantly increased at the acute stage, but decreased in the recurrence phase (Supplementary Figure 3). Similar to previous studies, parasitemia in μMT mice was elevated at day 10 post-infection, and mice could not clear the parasite and succumbed to infection (Figure 3E). To determine the role B cells play in the control of recurrence of *P. chabaudi*, B cells were depleted with anti-CD220 and anti-CD19 (Supplementary Figure 1). However, no significant difference in parasitemia was found between mice depleted of B cells and mice with B cells (Figure 3F). Overall, B cells thus do not play a role in the control of the parasitemic recurrence of *P. chabaudi*, which was also supported by the declined plasma cells during the recurrence stage.

### No Significant Effect of CD8^+^ T Cells on the Acute or Recurrent Phase of Infection

**FIGURE 4:**
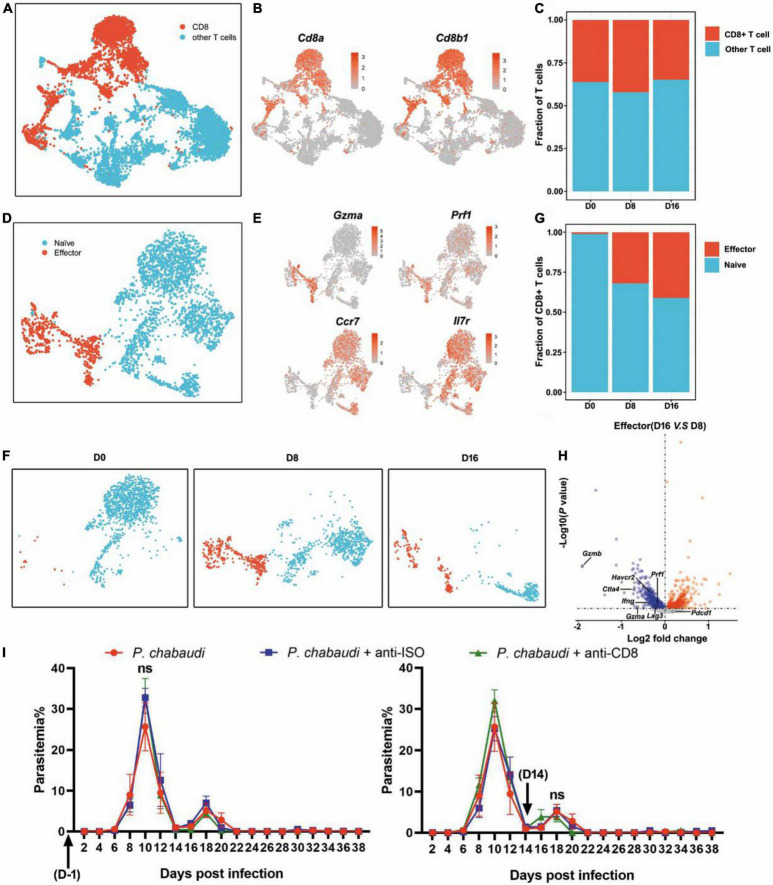
CD8^+^ T cells are dispensable for control of both the acute and recurrent stage of *P. chabaudi* infection. **(A)** UMAP projection plot of T cell populations merged from the three time points. **(B)** Feature plots depicting single-cell expression of canonical immune cell markers of CD8^+^ T cells. **(C)** Cell abundance frequency of CD8^+^ T cell relative to the total number of T cells at each time point. **(D)** UMAP visualization of the CD8^+^ T cells merged from all samples. **(E)** Expression of markers driving heterogeneity of CD8^+^ T cells subtypes, including the naïve and effector CD8^+^ T cells. **(F)** UMAP plot from **(D)** split into the three stages of infection. **(G)** Abundance of each population relative to the total number of CD8^+^ T cells at each time point. **(H)** Volcano plots depict differential gene expression between clusters of cells, with a *P*-value of < 0.05. Blue represents downregulated genes and red represents upregulated genes. **(I)** WT mice (*n* = 5) were injected with anti-CD8 or control IgG on days –1, 1, 3, 5, 7 or days 14, 16, 18, 20, 22 after the challenge. All experiments were performed in triplicates and the data are presented as means ± the SD. ns, not significant.

Recently, parasite specific CD8^+^ T cells were demonstrated to recognize and destroy reticulocytes infected with *P. vivax* and cooperate with macrophages to protect against blood-stage rodent malaria (Imai et al., 2015). However, the role of CD8^+^ T cells during *P. chabaudi* infection is still unknown. UMAP analysis showed that effector CD8^+^ T cells (*Cd3e, Cd8a, Cd8b1, Gzma, Prf1*) were increased in the acute phase and further increased in the recurrence phase (Figures 4A–G). Differential gene expression analysis also found that the signal of T cell exhausting markers, such as CTLA-4 (*Ctla4*) and Tim3 (*Harvcr2*), as well as granzyme B (*Gzmb*) and perforin (*Prf1*) were significantly downregulated in effector CD8^+^ T cells at the recurrence stage, as compared to the acute infection stage (Figure 4H). No significant effect on parasitemia was observed after CD8^+^ T cells were depleted during the acute or recurrence stage (Figure 4I). Thus, in contrast to its protective effect against *Plasmodium yoelii* (*P. yoelii*) infection, CD8^+^ T cells had no significant role in the control of either acute or recurrent *P. chabaudi* infection, which might be attributed to the exhaustion and dysfunction of CD8^+^ T cells during malaria infection (Horne-Debets et al., 2013).

### Innate Immune Responses Are Critical for the Control of *Plasmodium chabaudi* Recurrence

**FIGURE 5:**
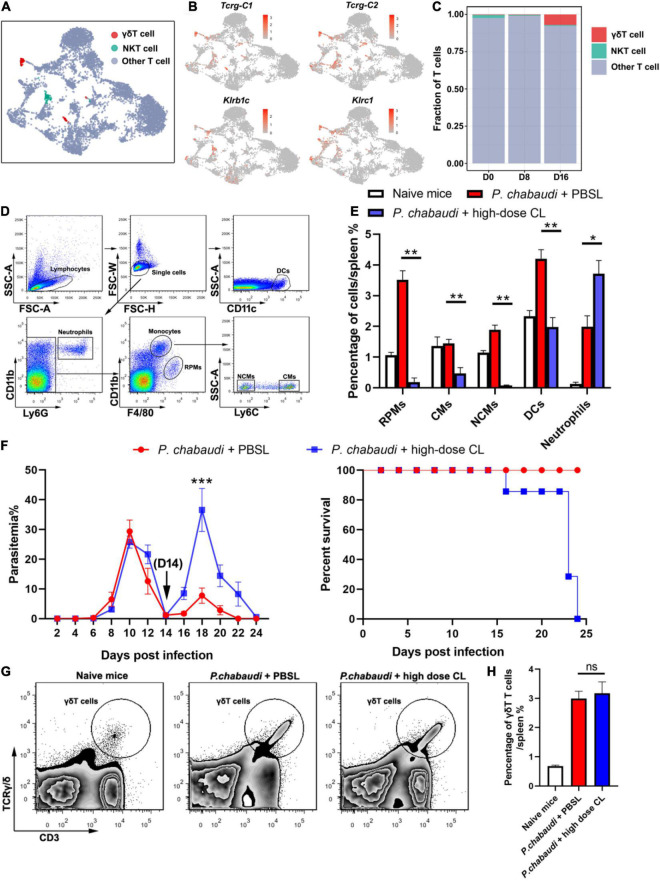
Innate immune cells are essential for the control of *P. chabaudi* recurrence. **(A)** UMAP projection plot of T cell populations merged from the three time points. **(B)** Feature plots depicting single-cell expression of canonical immune cell markers of γδT cells and NKT cells. **(C)** Cell abundance frequency of γδT cells and NKT cells relative to the total number of T cells at each time point. **(D)** A representative flow cytometry analysis of phagocytic cells, including the RPMs, CMs (classical monocytes), NCMs (non-classical monocytes), DCs and neutrophils. **(E)** Statistical analysis of the frequency of phagocytic cells in infected mice after treatment with CLs for 48 h. **(F)** WT mice (*n* = 5) were treated with clodronate liposomes (CLs) or control liposomes (PBSLs) on days 14, 17, 20 after the challenge. All the mice were challenged with *P. chabaudi* on day 0. The parasitemia (*left*) and survival rate (*right*) were monitored. **(G)** A representative flow cytometry analysis of γδT cells. **(H)** Statistical analysis of the frequency of γδT cells in mice after treatment with CLs for 48 h. Three individual experiments were performed. The data are presented as means ± the SD. ns, not significant; *P < 0.05; **P < 0.01; ***P < 0.001.

Based on the results obtained for the role of CD4^+^ T and B cells in the control of parasite recurrence in this study, we wondered whether the control of *P. chabaudi* recurrence was dependent on an innate immune response. UMAP analysis showed that the frequency of innate immune cells, including γδT cells, RPMs, DCs, and neutrophils, were significantly higher in the recurrence phase than in the acute phase (Figures 1E, 5A–C).

As a high dose of clodronate liposome (CL) has been shown to deplete most phagocytic cells in spleen tissue (Fontana et al., 2016), the *P. chabaudi* infected mice were administered a high dose of CL at the onset of the recurrent infection stage. As a result, almost 100% of the RPMs (CD11b^+^ F4/80^high^), 80% of the monocytes (CD11b^+^ F4/80^low^), and approximately 30% of the DCs (CD11c^+^), were depleted after the injection of CL. However, no neutrophil depletion was observed (Figures 5D,E). The parasitemia in the recurrent phase sharply increased to a level higher than the peak of the acute phase, and all mice died after treatment with CL (Figure 5F). Recently, γδT cells were reported to kill malaria parasites through direct phagocytosis (Junqueira et al., 2021). However, phagocytosis by γδT cells might result in its depletion by high-dose CL treatment. In contrast, we found that high-dose CL treatment did not reduce the frequency of γδT cells in the mice spleen (Figures 5G,H), therefore excluding the possibility that γδT cell depletion plays a role in parasitemia in mice treated with CL. Thus, our data strongly suggest the critical role of innate immune responses in the control the *P. chabaudi* recurrence.

## Discussion

Many studies have been conducted to elucidate the protective immune responses against acute malaria infection; however, knowledge about host control of parasite recrudescence is still lacking. In the current study, we analyzed the difference in host immune responses against acute infection and the recurrence of *P. chabaudi* infection. Our results illustrate the critical role of innate immune responses in the control of parasitemia recurrence when the adaptive immune responses are suppressed. These findings provide novel clues to immune intervention in the recrudescence of parasites and may aid in developing strategies to block malaria transmission.

Except for antigenic variation, the suppressive effect of malaria parasites on host immune responses is regarded as a major cause of parasite recrudescence (Butler et al., 2012; Horne-Debets et al., 2013). CD4^+^ T cell responses are the predominant protective immune response that controls blood-stage malaria infection. The differentiation of CD4^+^ T cells into Th1 and Tfh, which destroy the parasites through the secretion of IFN-γ or by helping B cells to produce high-affinity antibodies, has been observed in acute malaria infection (Stephens et al., 2005; Lonnberg et al., 2017; Perez-Mazliah et al., 2017). Meanwhile, Treg cells have been found to also increase early during malaria infection which limits the responses of Th1 and Tfh (Hisaeda et al., 2004; Nie et al., 2007; Haque et al., 2010). Accordingly, we found that Th1 and Tfh, along with Treg cells, increased early during acute *P. chabaudi* infection (Figure 2).

It has also been found that acute malaria infection impairs the Th1 response by inducing type I IFN secretion (Haque et al., 2014; Montes de Oca et al., 2016) and interferes with the differentiation of Tfh cells (Obeng-Adjei et al., 2015; Ryg-Cornejo et al., 2016). Furthermore, CD4^+^ T cells in chronic malaria infection tend to be exhausted, and their roles in the control of chronic malaria infection are greatly inhibited (Butler et al., 2012; Illingworth et al., 2012). In the current study, we observed a significant decrease in the number of Th1 cells and the expression of IFN-γ by Th1 in the recurrence phase of *P. chabaudi* infection. Additionally, our results demonstrated a less important role of CD4^+^ T cells in the control of parasite recurrence (Figure 2).

The antibodies produced by B cells require the help of Tfh cells. In the current study, the impaired Tfh response subsequently resulted in the inhibition of GC B cells activation, plasma cells, and antibody generation (Deenick and Ma, 2011). As a result, malaria infection resulted in an increase of atypical memory B cells (Weiss et al., 2009; Illingworth et al., 2012; Obeng-Adjei et al., 2017) and the production of antibodies was significantly suppressed (Portugal et al., 2015). Recently, *Plasmodium*-specific atypical memory B cells were regarded as short-lived activated B cells that disappeared upon natural resolution of chronic infection (Perez-Mazliah et al., 2018). In addition, malaria infection promotes the rapid development of plasmablasts, which results in nutrient deprivation of the germinal center reaction and limits the generation of memory B cells and LLPC responses (Vijay et al., 2020). In this study, we found that the frequency of plasma cells declined almost to baseline level in the recurrence phase, although the number of GC B cells and plasma cells were significantly increased during the acute *P. chabaudi* infection phase. This may indicate that plasma cells undergo apoptosis during the recurrence phase of *P. chabaudi* infection, as previously reported (Wykes et al., 2005).

With the suppressed functions of both CD4^+^ T and B cell responses, innate immune responses play a critical role in the control of parasite recurrence. Recently, a subset of γδT cells, Vδ2 T cells, was shown to be critical for the control of parasite recrudescence through the secretion of M-CSF (Mamedov et al., 2018), which in turn regulates the number of monocytes and macrophages (Ushach and Zlotnik, 2016). However, in addition to Vδ2 T cells, we found that RPMs, DCs and neutrophils were also greatly increased during recurrence phase of *P. chabaudi* infection. The increased number of macrophages and DCs might be differentiated from inflammatory monocytes recruited from the bone marrow during malaria infection, as previously reported (Lai et al., 2018). The depletion of DCs, monocytes, and RPMs by high dose CL treatment led to high levels of parasitemia during the recurrence phase, indicating their critical role in the control of parasite recrudescence. This effect was not attributed to the depletion of γδT cells, as high dose CL treatment did not lead to any loss of γδT cells in the spleen (Figures 5G,H). However, a detailed study is still needed to identify the individual roles of DCs and RPMs in the control of *P. chabaudi* recurrence.

With frequent recrudescence, human malaria infection persists for several months or years and serves as a major reservoir for malaria transmission (Okell et al., 2012; Churcher et al., 2015; Lin et al., 2016; Slater et al., 2019; Barry et al., 2021). If our finding that innate immune responses were critical for the control of parasite recrudescence could be confirmed in human malaria, it will help us to design novel strategy to control parasite recrudescence and to block malaria transmission.

## Data Availability Statement

The scRNA-seq data generated in this study have been deposited in the National Center for Biotechnology Information (NCBI) Gene Expression Omnibus (GEO) database at www.ncbi.nlm.nih.gov/geo/ under accession number GSE192930.

## Ethics Statement

The animal study was reviewed and approved by the Animal Ethics Committee of the Army Medical University (Third Military Medical University) Institute of Medical Research, under the approval number AMUWEC2020135.

## Author Contributions

WX, TL, and JZ: conceptualization, writing, review, editing, and supervision. WX and TL: writing – original draft and funding acquisition. TL, JZ, and SC: methodology. TL, SC, JZ, YG, YF, and SG: investigation. WX: resources. All authors contributed to the article and approved the submitted version.

## Conflict of Interest

The authors declare that the research was conducted in the absence of any commercial or financial relationships that could be construed as a potential conflict of interest.

## Publisher’s Note

All claims expressed in this article are solely those of the authors and do not necessarily represent those of their affiliated organizations, or those of the publisher, the editors and the reviewers. Any product that may be evaluated in this article, or claim that may be made by its manufacturer, is not guaranteed or endorsed by the publisher.

## Supplementary Material

Supplementary Figure 1 Depletion of selected cellular subsets during *P. chabaudi* infection. Cellular subsets were depleted by administering the depleting antibody i.p. every 2 days. Depletions included CD4^+^ T cells with anti-CD4, B cells with anti-CD19 and anti-B220, CD8^+^ T cells with anti-CD8β. The deletions were determined by flow cytometry, and the representative flow cytometry of the depletion of CD4^+^ T cells (*left*), B cells (*middle*) and CD8^+^ T cells (*right*) was presented.

Supplementary Figure 2 The identification of μMT mice by PCR. The μMT mice can be distinguished from the WT mice by PCR on genomic DNAs extracted from tails (WT mice = 210 bp, B^–/–^ mice = 500 bp, heterozygote mice = 210 + 500 bp), using the primers (OIMR1750: 5′-CCGTCTAGCTTGAGCTATTAGG-3′, OIMR1751: 5′-GAAGAG GACGATGAAGGTGG-3′, and OIMR5255: 5′-TTGTGCCCAGTCATAGCCGAAT -3′).

Supplementary Figure 3 Boxplot of expression levels of antibody subclasses genes in B cells at the three time points. The B cell cluster was further analyzed with the expression levels of antibody subclasses genes, including the *Ighm* (IgM), *Ighg2b* (IgG2b), *Ighg2c* (IgG2c), and *Ighg3* (IgG3). *P*-values in boxplots were calculated using two-sided Wilcoxon rank-sum test (^***^*P* < 0.001).

Supplementary Table 1 The differentially expressed genes of Th1 cells between day 16 and day 8 post-infection.

Supplementary Table 2 The differentially expressed genes of Treg cells between day 16 and day 8 post-infection.

Supplementary Table 3 The differentially expressed genes of effector CD8^+^ T cells between day 16 and day 8 post-infection.

## References

[B1] Barry A. Bradley J. Stone W. Guelbeogo M. W. Lanke K. Ouedraogo A. (2021). Higher gametocyte production and mosquito infectivity in chronic compared to incident *Plasmodium falciparum* infections. *Nat. Commun.* 12:2443. 10.1038/s41467-021-22573-7 33903595 PMC8076179

[B2] Brugat T. Reid A. J. Lin J. W. Cunningham D. Tumwine I. Kushinga G. (2017). Antibody-independent mechanisms regulate the establishment of chronic *Plasmodium* infection. *Nat. Microbiol.* 2:16276. 10.1038/nmicrobiol.2016.276 28165471 PMC5373435

[B3] Butler N. S. Moebius J. Pewe L. L. Traore B. Doumbo O. K. Tygrett L. T. (2012). Therapeutic blockade of PD-L1 and LAG-3 rapidly clears established blood-stage *Plasmodium* infection. *Nat. Immunol.* 13 188–195. 10.1038/ni.2180 22157630 PMC3262959

[B4] Chua C. L. Brown G. Hamilton J. A. Rogerson S. Boeuf P. (2013). Monocytes and macrophages in malaria: Protection or pathology? *Trends Parasitol.* 29 26–34. 10.1016/j.pt.2012.10.002 23142189

[B5] Churcher T. S. Trape J. F. Cohuet A. (2015). Human-to-mosquito transmission efficiency increases as malaria is controlled. *Nat. Commun.* 6:6054. 10.1038/ncomms7054 25597498 PMC4309425

[B6] Cohen S. Mc G. I. Carrington S. (1961). Gamma-globulin and acquired immunity to human malaria. *Nature* 192 733–737. 10.1038/192733a0 13880318

[B7] Deenick E. K. Ma C. S. (2011). The regulation and role of T follicular helper cells in immunity. *Immunology* 134 361–367. 10.1111/j.1365-2567.2011.03487.x 22043829 PMC3230790

[B8] Fontana M. F. de Melo G. L. Anidi C. Hamburger R. Kim C. Y. Lee S. Y. (2016). Macrophage colony stimulating factor derived from cd4+ t cells contributes to control of a blood-borne infection. *PLoS Pathog.* 12:e1006046. 10.1371/journal.ppat.1006046 27923070 PMC5140069

[B9] Haque A. Best S. E. Amante F. H. Mustafah S. Desbarrieres L. de Labastida F. (2010). CD4+ natural regulatory T cells prevent experimental cerebral malaria via CTLA-4 when expanded *in vivo*. *PLoS Pathog.* 6:e1001221. 10.1371/journal.ppat.1001221 21170302 PMC3000360

[B10] Haque A. Best S. E. Montes de Oca M. James K. R. Ammerdorffer A. Edwards C. L. (2014). Type I IFN signaling in CD8- DCs impairs Th1-dependent malaria immunity. *J. Clin. Invest.* 124 2483–2496. 10.1172/JCI70698 24789914 PMC4038565

[B11] Hisaeda H. Maekawa Y. Iwakawa D. Okada H. Himeno K. Kishihara K. (2004). Escape of malaria parasites from host immunity requires CD4+ CD25+ regulatory T cells. *Nat. Med.* 10 29–30. 10.1038/nm975 14702631

[B12] Horne-Debets J. M. Faleiro R. Karunarathne D. S. Liu X. Q. Lineburg K. E. Poh C. M. (2013). PD-1 dependent exhaustion of CD8+ T cells drives chronic malaria. *Cell Rep.* 5 1204–1213. 10.1016/j.celrep.2013.11.002 24316071

[B13] Illingworth J. Butler N. S. Roetynck S. Mwacharo J. Pierce S. K. Bejon P. (2012). Chronic exposure to *Plasmodium falciparum* is associated with phenotypic evidence of B and T cell exhaustion. *J. Immunol.* 190 1038–1047. 10.4049/jimmunol.1202438 23264654 PMC3549224

[B14] Imai T. Ishida H. Suzue K. Taniguchi T. Okada H. Shimokawa C. (2015). Cytotoxic activities of CD8 T cells collaborate with macrophages to protect against blood-stage murine malaria. *eLife* 4:e04232. 10.7554/eLife.04232 25760084 PMC4366679

[B15] Jagannathan P. Kim C. C. Greenhouse B. Nankya F. Bowen K. Eccles-James I. (2014). Loss and dysfunction of Vdelta2(+) gammadelta T cells are associated with clinical tolerance to malaria. *Sci. Transl. Med.* 6:251ra117. 10.1126/scitranslmed.3009793 25163477 PMC4198150

[B16] Julien J. P. Wardemann H. (2019). Antibodies against *Plasmodium falciparum* malaria at the molecular level. *Nat. Rev. Immunol.* 19 761–775. 10.1038/s41577-019-0209-5 31462718

[B17] Junqueira C. Barbosa C. R. R. Costa P. A. C. Teixeira-Carvalho A. Castro G. Sen Santara S. (2018). Cytotoxic CD8(+) T cells recognize and kill *Plasmodium vivax*-infected reticulocytes. *Nat. Med.* 24 1330–1336. 10.1038/s41591-018-0117-4 30038217 PMC6129205

[B18] Junqueira C. Polidoro R. B. Castro G. Absalon S. Liang Z. Sen Santara S. (2021). Gammadelta T cells suppress *Plasmodium falciparum* blood-stage infection by direct killing and phagocytosis. *Nat. Immunol.* 22 347–357. 10.1038/s41590-020-00847-4 33432229 PMC7906917

[B19] Lai S. M. Sheng J. Gupta P. Renia L. Duan K. Zolezzi F. (2018). Organ-specific fate, recruitment, and refilling dynamics of tissue-resident macrophages during blood-stage malaria. *Cell Rep.* 25 3099–3109.e3. 10.1016/j.celrep.2018.11.059 30540942

[B20] Lin J. T. Ubalee R. Lon C. Balasubramanian S. Kuntawunginn W. Rahman R. (2016). Microscopic *Plasmodium falciparum* gametocytemia and infectivity to mosquitoes in cambodia. *J. Infect. Dis.* 213 1491–1494. 10.1093/infdis/jiv599 26667316 PMC4813737

[B21] Lonnberg T. Svensson V. James K. R. Fernandez-Ruiz D. Sebina I. Montandon R. (2017). Single-cell RNA-seq and computational analysis using temporal mixture modelling resolves Th1/Tfh fate bifurcation in malaria. *Sci. Immunol.* 2:eaal2192. 10.1126/sciimmunol.aal2192 28345074 PMC5365145

[B22] Mamedov M. R. Scholzen A. Nair R. V. Cumnock K. Kenkel J. A. Oliveira J. H. M. (2018). A macrophage colony-stimulating-factor-producing gammadelta t cell subset prevents malarial parasitemic recurrence. *Immunity* 48 350–363.e7. 10.1016/j.immuni.2018.01.009 29426701 PMC5956914

[B23] Meding S. J. Langhorne J. (1991). CD4+ T cells and B cells are necessary for the transfer of protective immunity to *Plasmodium chabaudi chabaudi*. *Eur. J. Immunol.* 21 1433–1438. 10.1002/eji.1830210616 1675172

[B24] Montes de Oca M. Kumar R. Rivera F. L. Amante F. H. Sheel M. Faleiro R. J. (2016). Type I interferons regulate immune responses in humans with blood-Stage *Plasmodium falciparum* infection. *Cell Rep.* 17 399–412. 10.1016/j.celrep.2016.09.015 27705789 PMC5082731

[B25] Nie C. Q. Bernard N. J. Schofield L. Hansen D. S. (2007). CD4+ CD25+ regulatory T cells suppress CD4+ T-cell function and inhibit the development of *Plasmodium berghei*-specific TH1 responses involved in cerebral malaria pathogenesis. *Infect. Immun.* 75 2275–2282. 10.1128/IAI.01783-06 17325053 PMC1865737

[B26] Obeng-Adjei N. Portugal S. Holla P. Li S. Sohn H. Ambegaonkar A. (2017). Malaria-induced interferon-gamma drives the expansion of Tbethi atypical memory B cells. *PLoS Pathog.* 13:e1006576. 10.1371/journal.ppat.1006576 28953967 PMC5633206

[B27] Obeng-Adjei N. Portugal S. Tran T. M. Yazew T. B. Skinner J. Li S. (2015). Circulating Th1-cell-type Tfh cells that exhibit impaired b cell help are preferentially activated during acute malaria in children. *Cell Rep.* 13 425–439. 10.1016/j.celrep.2015.09.004 26440897 PMC4607674

[B28] Okell L. C. Bousema T. Griffin J. T. Ouedraogo A. L. Ghani A. C. Drakeley C. J. (2012). Factors determining the occurrence of submicroscopic malaria infections and their relevance for control. *Nat. Commun.* 3:1237. 10.1038/ncomms2241 23212366 PMC3535331

[B29] Ouedraogo A. L. Bousema T. Schneider P. de Vlas S. J. Ilboudo-Sanogo E. Cuzin-Ouattara N. (2009). Substantial contribution of submicroscopical *Plasmodium falciparum* gametocyte carriage to the infectious reservoir in an area of seasonal transmission. *PLoS One* 4:e8410. 10.1371/journal.pone.0008410 20027314 PMC2793432

[B30] Perez-Mazliah D. Gardner P. J. Schweighoffer E. McLaughlin S. Hosking C. Tumwine I. (2018). *Plasmodium*-specific atypical memory B cells are short-lived activated B cells. *eLife* 7:e39800. 10.7554/eLife.39800 30387712 PMC6242553

[B31] Perez-Mazliah D. Nguyen M. P. Hosking C. McLaughlin S. Lewis M. D. Tumwine I. (2017). Follicular helper t cells are essential for the elimination of *Plasmodium* Infection. *EBioMedicine* 24 216–230. 10.1016/j.ebiom.2017.08.030 28888925 PMC5652023

[B32] Pombo D. J. Lawrence G. Hirunpetcharat C. Rzepczyk C. Bryden M. Cloonan N. (2002). Immunity to malaria after administration of ultra-low doses of red cells infected with *Plasmodium falciparum*. *Lancet* 360 610–617. 10.1016/S0140-6736(02)09784-2 12241933

[B33] Portugal S. Tipton C. M. Sohn H. Kone Y. Wang J. Li S. (2015). Malaria-associated atypical memory B cells exhibit markedly reduced B cell receptor signaling and effector function. *eLife* 4:e07218. 10.7554/eLife.07218 25955968 PMC4444601

[B34] Ryg-Cornejo V. Ioannidis L. J. Ly A. Chiu C. Y. Tellier J. Hill D. L. (2016). Severe malaria infections impair germinal center responses by inhibiting t follicular helper cell differentiation. *Cell Rep.* 14 68–81. 10.1016/j.celrep.2015.12.006 26725120

[B35] Scherf A. Lopez-Rubio J. J. Riviere L. (2008). Antigenic variation in *Plasmodium falciparum*. *Annu. Rev. Microbiol.* 62 445–470. 18785843 10.1146/annurev.micro.61.080706.093134

[B36] Slater H. C. Ross A. Felger I. Hofmann N. E. Robinson L. Cook J. (2019). The temporal dynamics and infectiousness of subpatent *Plasmodium falciparum* infections in relation to parasite density. *Nat. Commun.* 10:1433. 10.1038/s41467-019-09441-1 30926893 PMC6440965

[B37] Stephens R. Albano F. R. Quin S. Pascal B. J. Harrison V. Stockinger B. (2005). Malaria-specific transgenic CD4(+) T cells protect immunodeficient mice from lethal infection and demonstrate requirement for a protective threshold of antibody production for parasite clearance. *Blood* 106 1676–1684. 10.1182/blood-2004-10-4047 15890689

[B38] Stephens R. Culleton R. L. Lamb T. J. (2012). The contribution of *Plasmodium chabaudi* to our understanding of malaria. *Trends Parasitol.* 28 73–82. 10.1016/j.pt.2011.10.006 22100995 PMC4040349

[B39] Ushach I. Zlotnik A. (2016). Biological role of granulocyte macrophage colony-stimulating factor (GM-CSF) and macrophage colony-stimulating factor (M-CSF) on cells of the myeloid lineage. *J. Leukoc. Biol.* 100 481–489. 10.1189/jlb.3RU0316-144R 27354413 PMC4982611

[B40] Vijay R. Guthmiller J. J. Sturtz A. J. Surette F. A. Rogers K. J. Sompallae R. R. (2020). Infection-induced plasmablasts are a nutrient sink that impairs humoral immunity to malaria. *Nat. Immunol.* 21 790–801. 10.1038/s41590-020-0678-5 32424361 PMC7316608

[B41] von der Weid T. Kitamura D. Rajewsky K. Langhorne J. (1994). A dual role for B cells in *Plasmodium chabaudi chabaudi (AS)* infection? *Res. Immunol.* 145 412–419. 10.1016/s0923-2494(94)80170-3 7899705

[B42] Weiss G. E. Crompton P. D. Li S. Walsh L. A. Moir S. Traore B. (2009). Atypical memory B cells are greatly expanded in individuals living in a malaria-endemic area. *J. Immunol.* 183 2176–2182. 10.4049/jimmunol.0901297 19592645 PMC2713793

[B43] Wykes M. N. Zhou Y. H. Liu X. Q. Good M. F. (2005). *Plasmodium yoelii* can ablate vaccine-induced long-term protection in mice. *J. Immunol.* 175 2510–2516. 10.4049/jimmunol.175.4.2510 16081823

