# Supplementary figures and images for: The Dynamic Change of Immune Responses Between Acute and Recurrence Stages of Rodent Malaria Infection
Source: Front Microbiol. 2022 Feb 17;13:844975. doi: 10.3389/fmicb.2022.844975 (PMC8891988; doi:10.3389/fmicb.2022.844975)

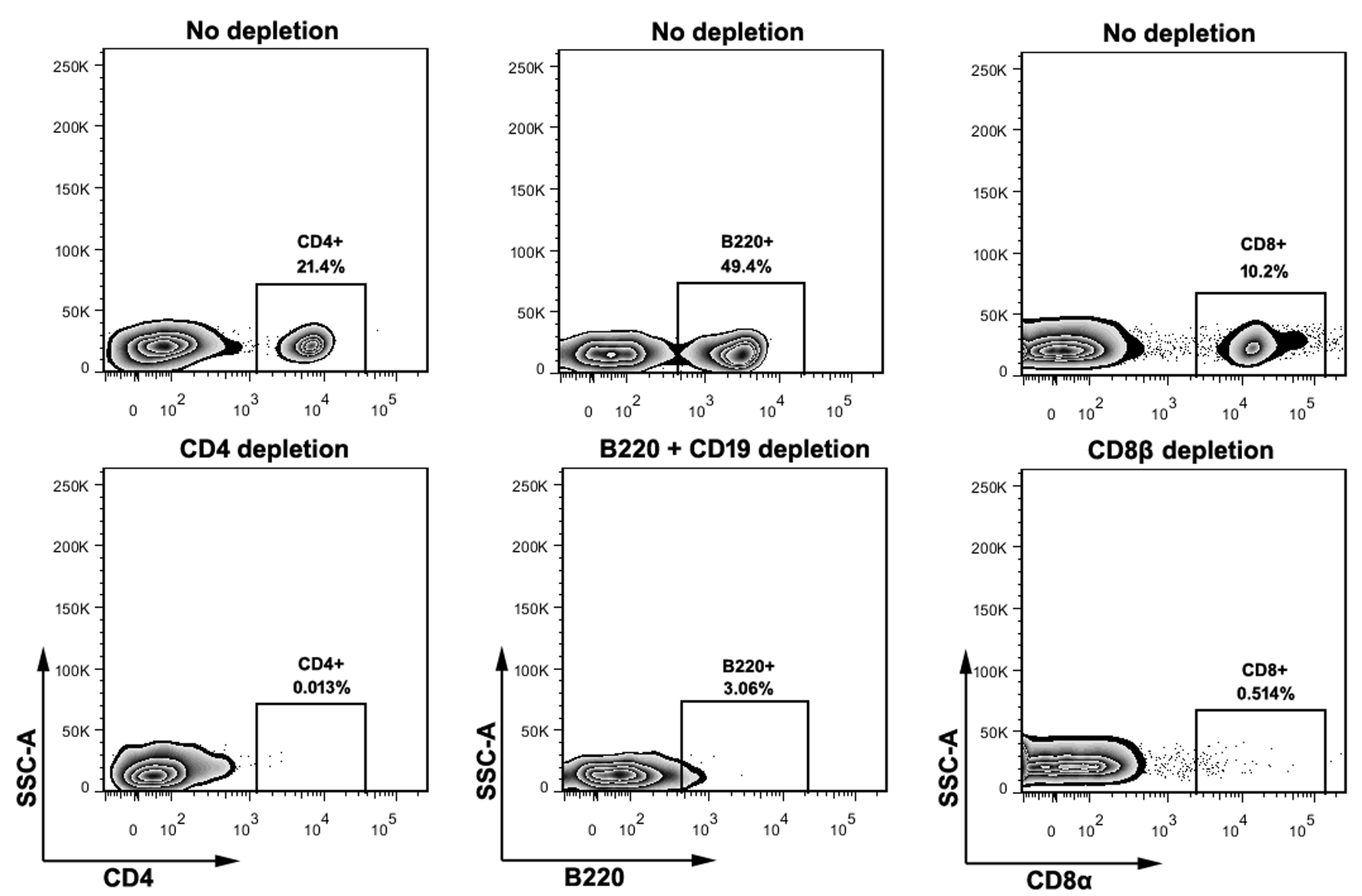

Supplement: Supplementary Figure 1 — Depletion of selected cellular subsets during P. chabaudi infection. Cellular subsets were depleted by administering the depleting antibody i.p. every 2 days. Depletions included CD4+ T cells with anti-CD4, B cells with anti-CD19 and anti-B220, CD8+ T cells with anti-CD8β. The deletions were determined by flow cytometry, and the representative flow cytometry of the depletion of CD4+ T cells (left), B cells (middle) and CD8+ T cells (right) was presented. [file Image_1.TIF]

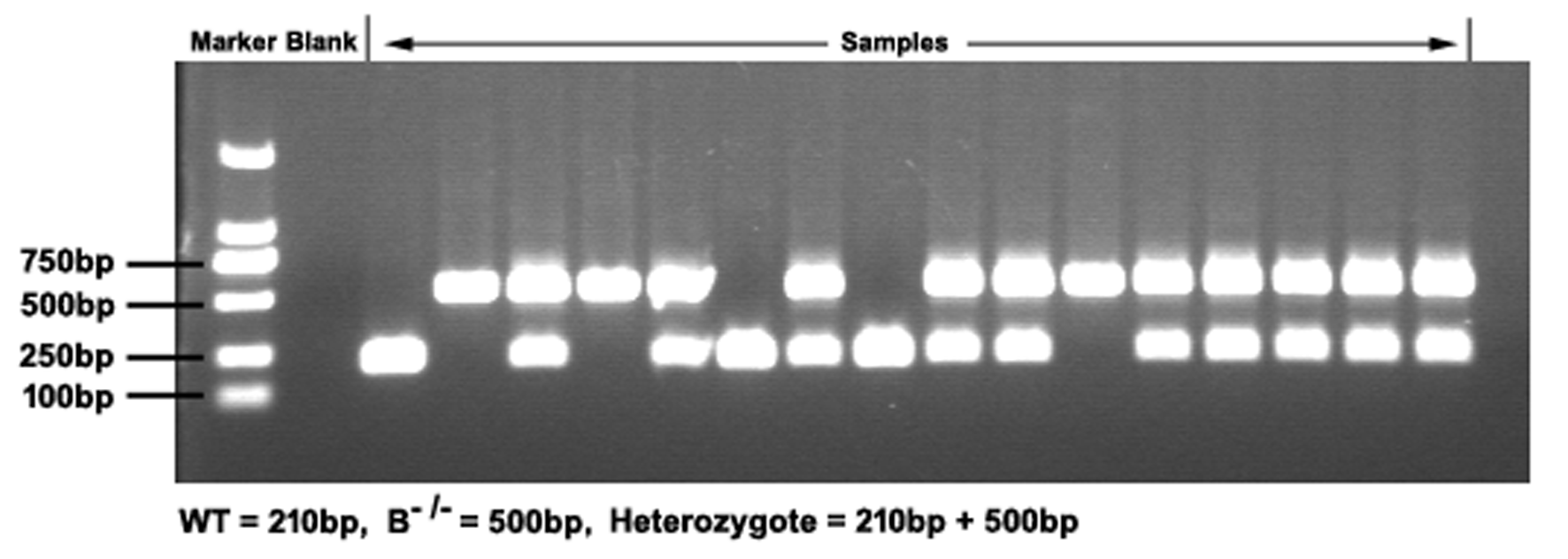

Supplement: Supplementary Figure 2 — The identification of μMT mice by PCR. The μMT mice can be distinguished from the WT mice by PCR on genomic DNAs extracted from tails (WT mice = 210 bp, B–/– mice = 500 bp, heterozygote mice = 210 + 500 bp), using the primers (OIMR1750: 5′-CCGTCTAGCTTGAGCTATTAGG-3′, OIMR1751: 5′-GAAGAG GACGATGAAGGTGG-3′, and OIMR5255: 5′-TTGTGCCCAGTCATAGCCGAAT -3′). [file Image_2.TIF]

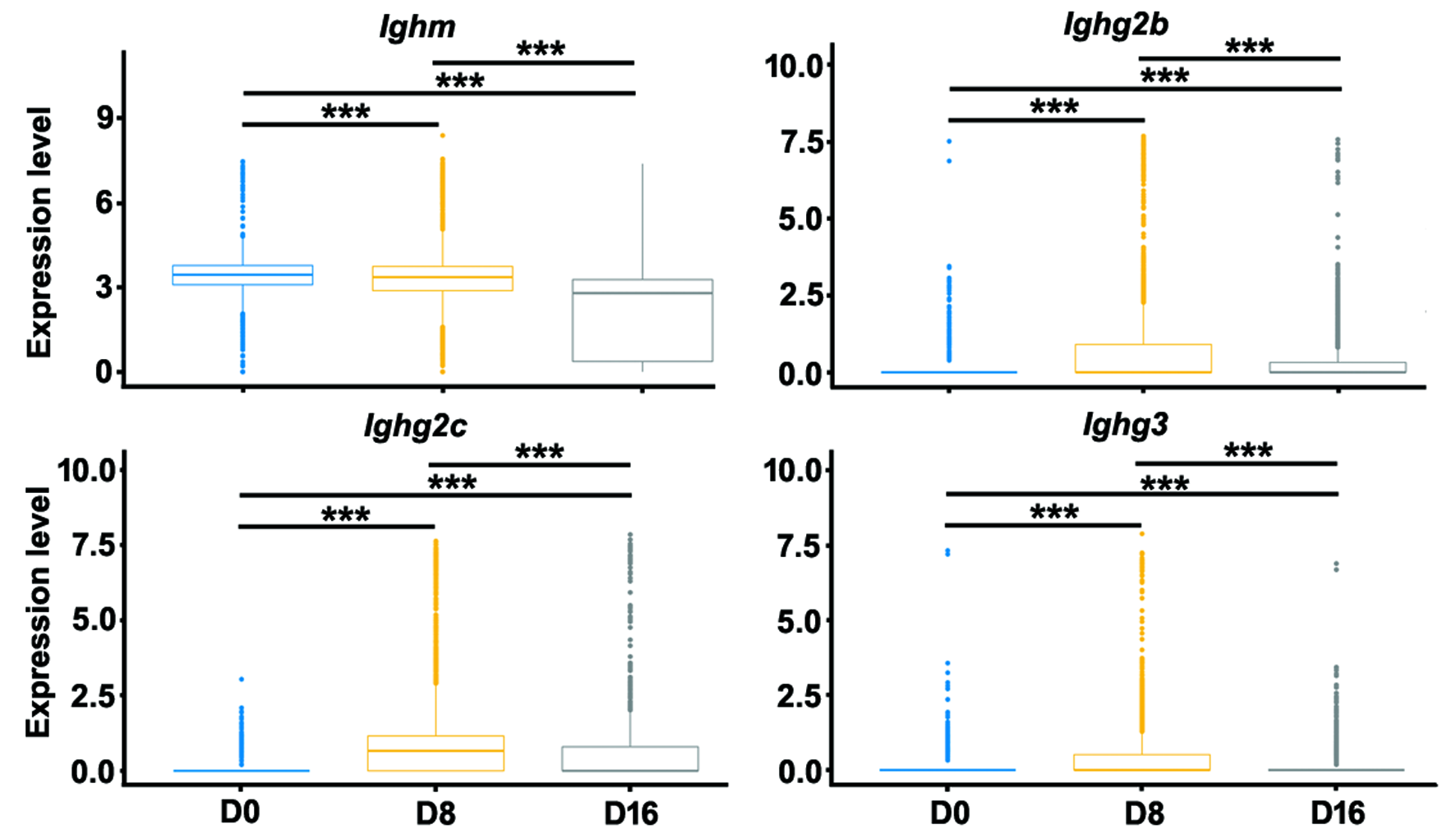

Supplement: Supplementary Figure 3 — Boxplot of expression levels of antibody subclasses genes in B cells at the three time points. The B cell cluster was further analyzed with the expression levels of antibody subclasses genes, including the Ighm (IgM), Ighg2b (IgG2b), Ighg2c (IgG2c), and Ighg3 (IgG3). P-values in boxplots were calculated using two-sided Wilcoxon rank-sum test (***P < 0.001). [file Image_3.TIF]
